# The time delay between *in vivo* imaging and *postmortem* data poses a caveat on “no link” findings

**DOI:** 10.1002/mds.27847

**Published:** 2019-10-18

**Authors:** Christian Pifl

**Affiliations:** ^1^ Center for Brain Research Medical University of Vienna Vienna Austria

I read with interest the article by Honkanen and colleagues^1^ on the question of correlation between striatal dopaminergic innervation and dopamine transporter (DAT) imaging as assessed by putamen tyrosine hydroxylase–positive axon counts and DAT single‐photon emission computed tomography (SPECT). In their 14 patients with neuropathologically confirmed Parkinson's disease or atypical parkinsonism, specific binding ratios (SBRs) from SPECT did not correlate with the total putamen tyrosine hydroxylase‐positive fiber counts. This is very surprising given that DATs are densely present on dopaminergic axons with varicosities,^2^, ^3^ and nearly all striatal tyrosine hydroxylase is contained in axons of the dopaminergic mesostriatal pathways.^4^

The key to the discrepancy between lack of correlation in Honkanen and colleagues and the well‐established cellular coexpression of DAT and tyrosine hydroxylase in the striatum may lay in the fact that a correlation was investigated between an *in vivo* (SBR) and a *postmortem* parameter (tyrosine hydroxylase–positive axon counts) with a considerable time interval in between. Obviously, a long interval between scan and death means an imaging analysis earlier in the disease process with still high SBRs for DAT and therefore lets one expect higher SBRs. Furthermore, a long interval between death and autopsy affects the tyrosine hydroxylase–positive axon counts: Impairment in immunohistochemical stains can be caused by postmortem delay attributed to autolysis and enzymatic activation.^5^ The individualization of the data, together with the individual intervals between scan and death and between death and autopsy in Honkanen and colleagues, enables the reader to make a correlation between the *in vivo* and *postmortem* findings and the two time intervals. And, in fact, it turns out that the data of SBRs and intervals between scan and death reveal a positive correlation coefficient of 0.371 with a *P* value of 0.184 (Fig. 1A), and omission of two cases results in a highly significant correlation of 0.713 with a *P* value of 0.008 (Fig. 1B; Spearman rank‐order correlation). Furthermore, as predicted, there is a negative correlation coefficient of –0.391 between tyrosine hydroxylase–positive fiber counts and interval between death and autopsy (varying between 1 and 8 days!) with a *P* value of 0.162 calculated for the 14 patients (Fig. 1C), and omission of just one case results in a significant correlation of –0.648 with a *P* value of 0.016 (Fig. 1D).

**Figure 1:**
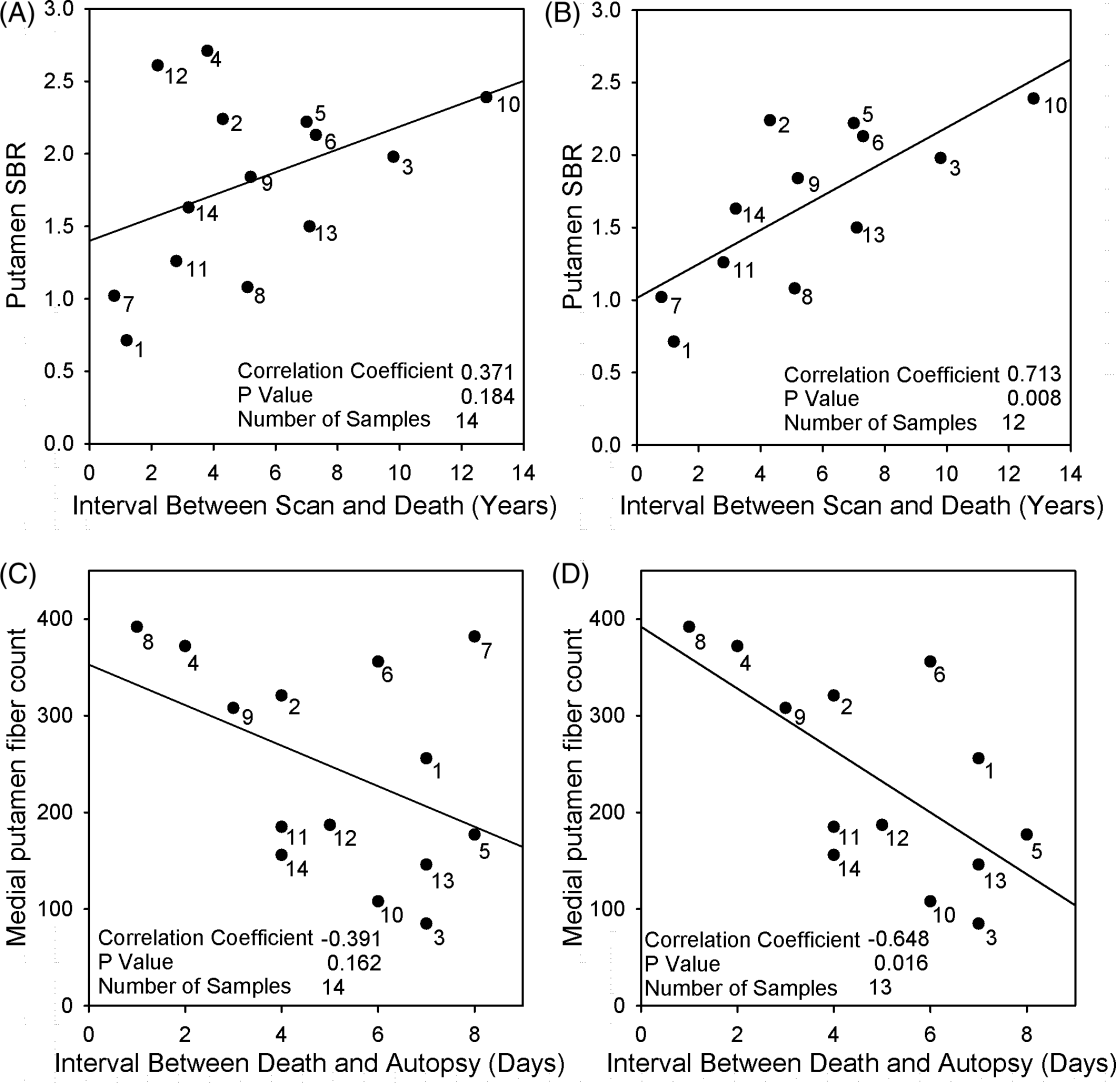
(A,B) Scatter plot presenting a positive correlation between the putamen SBR calculated on DAT SPECT data and the interval between the time of the respective scan and death for all 14 cases (A) and for 12 of these cases (B). (C,D) Scatter plot presenting a negative correlation between the medial putamen fiber counts and the interval between death and autopsy for all 14 cases (C) and for 13 of these cases (D).

If the two parameters, SBR and fiber counts, are biased to such a high extent by time variables, it is not surprising that no correlation was detected between them, and, for such a low number of cases, this problem cannot be resolved by calculations using death to autopsy as covariates. Therefore, the conclusion that striatal DAT imaging may not reflect striatal dopaminergic projections axons cannot be drawn from this study.

## Financial Disclosures

Nothing to report.
